# Metal-Containing Formate Dehydrogenases, a Personal View

**DOI:** 10.3390/molecules28145338

**Published:** 2023-07-11

**Authors:** Silke Leimkühler

**Affiliations:** Department of Molecular Enzymology, Institute of Biochemistry and Biology, University of Potsdam, Karl-Liebknecht-Str. 24–25, 14476 Potsdam, Germany; sleim@uni-potsdam.de; Tel.: +49-3319775603

**Keywords:** molybdoenzymes, bis-MGD, formate oxidation, CO_2_ reduction, oxygen atom transfer

## Abstract

Mo/W-containing formate dehydrogenases (FDH) catalyzes the reversible oxidation of formate to carbon dioxide at their molybdenum or tungsten active sites. The metal-containing FDHs are members of the dimethylsulfoxide reductase family of mononuclear molybdenum cofactor (Moco)- or tungsten cofactor (Wco)-containing enzymes. In these enzymes, the active site in the oxidized state comprises a Mo or W atom present in the bis-Moco, which is coordinated by the two dithiolene groups from the two MGD moieties, a protein-derived SeCys or Cys, and a sixth ligand that is now accepted as being a sulfido group. SeCys-containing enzymes have a generally higher turnover number than Cys-containing enzymes. The analogous chemical properties of W and Mo, the similar active sites of W- and Mo-containing enzymes, and the fact that W can replace Mo in some enzymes have led to the conclusion that Mo- and W-containing FDHs have the same reaction mechanism. Details of the catalytic mechanism of metal-containing formate dehydrogenases are still not completely understood and have been discussed here.

## 1. Introduction

**Figure 1:**
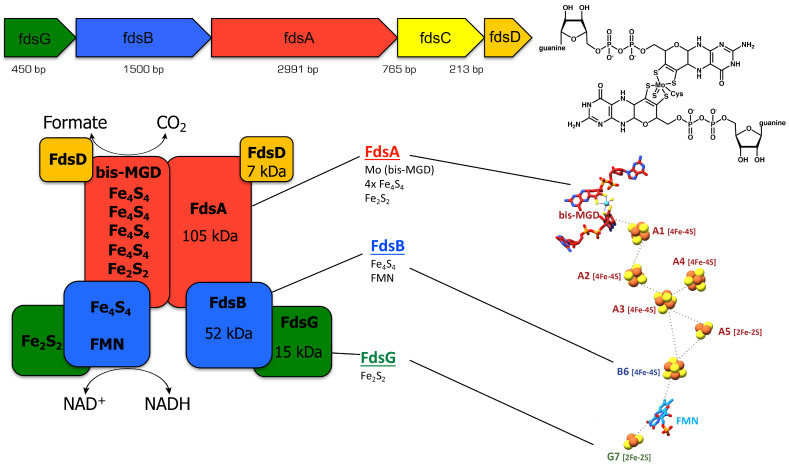
Schematic presentation of the *FdsGBACD* gene region, the bis-MGD cofactor, the subunit arrangement and the FeS clusters of *R. capsulatus* FDH. On the top, the *FdsGBACD* gene region and the bis-MGD cofactor of *R. capsulatus* FDH are shown, with the sulfido ligand and the Cys bis-MGD ligation. On the bottom, the subunit arrangement and the cofactors are shown from the cryo-EM structure (right part of the figure adapted from Figure 2 in publication [8], including the numbering of the FeS clusters in *R. capsulatus* FDH.

I have been working on this enzyme for 15 years now; is this enough time to provide a personal opinion? As I have been working in the field of molybdoenzymes since 1994, 30 years now, I accept that a personal opinion might be justified. It all started with the work on *Rhodobacter capsulatus* in 1994 in Bielefeld, when I started my PhD project in the laboratory of Werner Klipp. Werner was well established and worked on the genetic manipulation and regulation of genes in this bacterium. While searching for a PhD project for me, he came across xanthine dehydrogenase (XDH), since the idea was to work on a gene cluster that might be regulated by both Mo and nitrogen. The research started with a Tn5 mutagenesis screen to find the genes encoding XDH in this bacterium, which had not been sequenced at that time. We picked 40,000 colonies together with colleagues in his lab and indeed identified genes with Tn5 insertions encoding XDH or Moco biosynthesis enzymes [1]. This took quite some effort, which is much easier nowadays with all the genome sequences being readily available. I cloned the genes and looked at their regulation, which revealed no regulatory control of the *Xdh* operon with everything I tested. I then became interested in the enzyme, which was quite a challenge in a pure genetic lab. Thus, I was very happy when Al McEwan, a visitor from Australia, gave an invited talk on the DMSO reductase enzyme from *Rhodobacter capsulatus* [2] in 1995. He wanted his PhD students to learn about genetics using this organism. We rapidly came to the conclusion that a mutual exchange of students would be the best idea, for me to learn enzymology and protein purification in his lab and for his student to learn genetics in our lab. Therefore, I was lucky enough to travel to Australia in 1996 for 3 months during my Ph.D. thesis work. I had to send all the expression strains I had constructed ahead of time by regular mail, so I expected to start work immediately after my arrival. However, I was surprised to see that my precious strains were stored at 4 °C in the cold room, something we never did in Bielefeld, since it was believed that the strains would not survive these low temperatures. Lucky enough the strains were nevertheless growing and I could start my practical work with enzyme purification right away. I was introduced to classical protein purification (without any affinity tags) and started to grow large amounts of culture, since the enzyme was expected to be expressed in low amounts. I was growing 100 L of culture per week to obtain enough culture for the classical purification procedures (French press, ammonium sulfate precipitation) followed by various columns to be tested (anion exchange, phenyl sepharose, hydroxyl apatite, size exclusion). After 3 months of work, I succeeded in purifying enough enzyme to visualize on an SDS gel after silver staining. Anyway, I learned a lot and was able to establish protein purification back home in Bielefeld that, with some modifications, resulted in sufficient purification of the enzyme for UV–visible spectra, enzyme assays and Moco analysis [1]. During my following postdoc-time in the lab of KV Rajagopalan at Duke University, Durham, NC, USA, I established a system for the heterologous expression of the enzyme in *E. coli* [3]. Through the addition of an affinity-tag (6x His Tag), the purification was facilitated and the enzyme was pure enough for crystallization studies. The structure was later solved in collaboration with Caroline Kisker in 2001 [4]. Later, with Takeshi Nishino, we studied the inhibition of the enzyme with his synthesized inhibitors, and surprisingly, the enzyme was not inhibited by a very potent inhibitor of the human enzyme (febuxostat) [5]. Coming back to the collaboration with Al McEwan, the visit by the Australian PhD student in Germany also went well and we constructed a couple of *R. capsulatus* mutant strains in the genes for DMSO reductase together [6]. In Australia, I was also introduced to the work on DMSO reductase, the main focus of the lab, and to the awful smell of DMS that accompanies working with this enzyme. Nowadays, when I smell DMS, I am reminded of my time in Australia. Later, during my habilitation time (2001–2004) in the lab of Ralf Mendel in Braunschweig, I was mainly focusing on human Moco biosynthesis. However, in 2009, after I obtained my full Professor position at the University of Potsdam, I was writing a grant application on bacterial molybdoenzymes and I recalled my work on *Rhodobacter capsulatus*. I checked and found an uncharacterized molybdoenzyme in this bacterium: formate dehydrogenase (FDH). Since the grant application was funded, I started to work on this enzyme. Luckily, a very talented student, Tobias Hartmann, who had just finished his diploma thesis, was looking for a PhD position. I offered him the position on the formate dehydrogenase project and he was eager to accept this, since he was somehow fed up with his previous work on human aldehyde oxidase. He started to clone the *fdsGBACD* genes (Figure 1) and purify the proteins after heterologous expression was observed in *E. coli* in 2009, which resulted in the first publication on FDH from my lab in 2013 [7].

## 2. Facing the First Problems and New Challenges Working with FDH

Tobias started with the cloning of *R. capsulatus fdh* from genomic DNA and was facing the “normal” PCR problems with the very GC-rich DNA of *Rhodobacter*, so the cloning took a bit longer than expected. Tobias finally succeeded in expressing the enzyme in *E. coli*, adapting the expression system established for XDH, and the enzyme was purified as a dimer of heterotrimers comprising the FdsGBA subunits [7] (Figure 1). The big question was whether the FdsD protein is bound as a subunit to FdsGBA, so Tobias tried many approaches to make FdsD visible, but without success. Consequently, we concluded that it is not bound as a subunit to FdsGBA. This was in contrast to what had been observed for the homologous FDH from *Alcaligenes eutrophus* (now *Cupriavidus necator*) [9], but we had to believe our results at that time. During this time, we held our last “Molybdenum and Tungsten enzymes” conference as a Gordon Conference in Il Ciocco, Italy in 2009. One of the external attendees was enquiring as to whether Mo/W enzyme researchers would be interested in working on FDH. Since we has already started to work on *R. capsulatus* FDH, I also agreed to work on and reinvestigate *E. coli* FdhF, which I was eager to homologously express in *E coli*. All of the other attendees that this person spoke to at the conference refused to start working on this enzyme. This was on the basis that everything was known about this enzyme already, since the crystal structure of the *E. coli* enzyme had been solved in 1997 [10]. Nevertheless, I agreed to work on this enzyme, since I thought that there were still open research questions remaining and that the role in CO_2_ reduction might be of interest for future applications. I was quite shocked to learn of the reaction of my colleagues back then. Apparently, many of them changed their minds years later when they discovered that FDH is indeed an interesting enzyme to work on with challenging future perspectives. Therefore, nowadays, almost everyone in the field of molybdoenzymes is working on an FDH enzyme from various organisms; thus, competition in the field is very high. My group then additionally started to work on the *E. coli* FdhF enzyme and on its homologous expression in *E. coli*. However, since the enzyme contains a selenocysteine residue in the active site, which is crucial for its activity, the expression system was more challenging to establish than initially expected. We tried many approaches, including the co-expression of the selenocysteine synthesis machinery. In the end, we succeeded in obtaining the enzyme, but not with a high yield and still only with a poor selenocysteine content. To date, we have not published anything on this enzyme, but we have provided our expression clone to other researchers, including Judy Hirst [11] and John Golbeck.

## 3. The Role of the Sulfido Ligand in FDH

FDH had been expected to have a sulfido ligand bound at its active site, like the enzymes from the xanthine oxidase family [12]. The nature of the Mo ligand was not so clear in the first crystal structures of the *E. coli* [10,13] and *Desulfovibrio* [14] enzymes, whereas the presence of a sulfido ligand was clearly resolved in the formylmethanofuran dehydrogenase at a 1.9 A resolution [15]. Later, it was revealed by biochemical studies that the FdhD protein in *E. coli* and the FdsC protein in *R. capsulatus* are involved in the building of the sulfido ligand [16,17], with the sulfur originating from L-cysteine desulfurases like IscS [18]. My group confirmed the presence of the sulfido ligand using extended X-ray absorption fine structure (EXAFS) studies (in collaboration with Michael Haumann), which also showed that the ligand was absent in the absence of FdsC [19]. We further showed, for the first time, that FdsC binds bis-molybdopterin guanine dinucleotide (MGD) [20], which was later confirmed by crystallographic studies using *E. coli* FdhD [18]. What is the role of the sulfido ligand? While the ligand had been proposed by electron spin resonance (EPR) spectroscopic studies to be the acceptor of the hydride atom during catalysis [21], we further showed that it contributes to the oxygen sensitivity of the enzyme [13]. While most enzymes are stabilized by inhibitors to make them more oxygen stable, we revealed that the site of the oxygen sensitivity in the absence of an inhibitor is the sulfido ligand, that is easily replaced by oxygen when oxygen is present in the uninhibited enzyme. The inhibitor was shown to bind in the vicinity of the sulfido ligand, thereby preventing the access of oxygen to the active site so that the sulfido ligand remains in place [22]. Later, the Hille group published that the air inactivation is due to the production of superoxide by the *Cupriavidus necator* enzyme in the presence of oxygen [23]. In this case, superoxide produced at the flavin mononucleotide (FMN) cofactor reacts with the sulfido ligand, which produces sulfite and replaces the sulfido ligand with oxygen. However, this mechanism of inactivation can only be applied to (FMN)-containing enzymes that react with nicontinamide adenine dinucleotide (NAD^+^) or oxygen as electron acceptors, producing superoxide in the reaction with oxygen. Other FDH enzymes must have a different mechanism resulting in their oxygen sensitivity.

## 4. Cryo-EM Structure of FDH

Shortly before Tobias left the lab for a position in industry, he established a system for the homologous expression of *R. capsulatus* FDH in *R. capsulatus*, a system based on the expression of XDH that I had established during my PhD work. When the new postdoc, Gerd Mittelstaedt, started, a former diploma student who obtained his PhD in crystallography in New Zealand, he was using this system to purify FDH to try cryogenic electron microscopy (EM) with our new colleague, Petra Wendler at the University of Potsdam. Gerd was very lucky and they immediately obtained nice-looking data in the negative stain from his first purification. The purification was optimized once more and data sets were obtained with the oxidized formate and NADH-reduced enzymes, inhibited by azide. Gerd worked on solving the structure and he generated the first data sets, which were later refined by Petra after Gerd left the lab for a permanent position in New Zealand. The new insight from the cryo-EM structure was the presence of the FdsD subunit present as a cap on FdsA [8] (Figure 1). It seems likely that, most of the time, the subunit was not stained well in our SDS gels, so it was only “sometimes” visible after the purification of FDH, but was likely always present. Petra obtained data sets with the subtracted reduced and oxidized structures, in which the reduced cofactors were visible. To our surprise, all of the iron sulfur clusters were reduced in her subtracted data set, which did not fit with our results obtained by EPR (measured by the postdoc Benjamin Duffus in my lab), in which one cluster (likely the A4 cluster) was never reduced (Figure 1). Further, in the meantime, I was also asked to review a PhD thesis from the University of Denmark, with the topic of the comparison of *R. capsulatus, Rhodobacter sphaeroides* and *C. necator* FDH enzymes. I was very positively surprised and happy to read that a PhD student in a lab that had nothing to do with molybdoenzymes, was able to reproduce most of our results on the purification and kinetic characterization of *R. capsulatus* FDH. The only difference was that he was also able to purify the enzyme as an αβγ monomer, with the monomer being as active as the dimer [24]. This highlighted to me that there is no electron transfer between the dimers, since the *k*_cat_ had remained identical; results that were also consistent with what we had obtained. Overall, I was very happy to read that an independent person was able to reproduce our results, which demonstrated the reliability and reproducibility of our work.

## 5. Inhibition of FDHs by Small Molecules

The inhibition of different FDH enzymes has been studied intensively using various inhibitors; among them, the most prominent is azide. Azide is an isoelectronic molecule to CO_2_, which had been thought to be a transition state analogue for the FDH reaction, and is most often used during the purification of FDHs to protect the enzyme from oxidative damage and the loss of the sulfido ligand. In a study using the *E. coli* FdhF enzyme, a set of inhibitors of varying electron donor strengths (N_3_^−^, OCN^−^, SCN^−^, NO_2_^−^ and NO_3_^−^) was used to study their binding to the reduced and oxidized FdhF enzyme. Among these inhibitors, inhibition with N_3_^−^, NO_2_^−^ and NO_3_^−^ has also been studied using various FDH enzymes from other organisms. The inhibition studies revealed that inhibitor binding is oxidation-state-dependent, since different results were obtained by studying the inhibition mode of the reaction of formate oxidation versus the reverse reaction of CO_2_ reduction. While all inhibitors were more potent in terms of inhibiting the reaction of formate oxidation, azide and cyanate are generally reported to be mixed-type inhibitors, revealing two binding sites, with one being competitive for the substrate formate and the other binding site being non-competitive. NO_2_^−^ and NO_3_^−^ are generally competitive inhibitors that use formate as a substrate. In the crystal structure of *E. coli* FdhF, NO_2_^−^ was proposed to be coordinated with the Mo ion.

Robinson et al. [11] concluded that inhibitors like azide, cyanate and NO_3_ bind more tightly to a vacant coordination site on Mo^VI^ than to Mo^IV^, based on the oxidation state. It was further suggested that the SeCys residue in the *E. coli* enzyme has to dissociate from the Mo ion to generate a vacant position to which the inhibitors can bind. However, in a more recent study by the postdoc Benjamin Duffus in my group, in collaboration with Ingo Zebger and Konstantin Laun, who were investigating the inhibition of *R. capsulatus* FDH by azide and cyanate using infrared (IR) spectroscopy [22], it was revealed that neither inhibitor binds directly to the Mo ion in the reaction of formate oxidation. In this study, both inhibitors showed a mixed-type inhibition and two binding sites were identified by IR spectroscopy. The competitive and non-competitive azide binding site involves arginine in the second coordination sphere, and the former was also shown to be dependent on the presence of the bis-MGD cofactor. This suggested that the azide binds between the sulfido ligand of the bis-MGD cofactor and Arg587, which might also explain the increased oxygen stability of azide-inhibited enzymes by sterically shielding the ligand against oxidative damage. In contrast, the location of the non-competitive binding site could not be resolved in this study, but might very well involve the arginine residue in a bis-MGD-independent fashion [22]. A displacement of the cysteine ligand was not observed by IR spectroscopy. With azide being isoelectronic to CO_2_, the inhibition mode for the reaction of CO_2_ reduction needs to be investigated in more detail to shed light on the different inhibition modes and strengths of inhibition for the different oxidation states of the Mo ion. In conjunction with mechanistic aspects described in the following section, it also remains likely that formate and CO_2_ have different binding sites in the active site and that the inhibitors bind with a different affinity to these binding sites.

## 6. The Reaction Mechanism of FDHs

Overall, the metal-dependent enzymes are much better catalysts for the reaction of CO_2_ reduction, and for a long time it was believed that the metal-independent enzymes are not able to perform the back reaction overall [21]. The reaction mechanism for metal-independent FDHs was identified to be a classical hydride transfer mechanism, involving NAD^+^ at the active site [25]. The metal-dependent enzymes include Mo- or W-containing enzymes binding the bis-MGD cofactor [12]. While metal-dependent FDH enzymes have been studied for several decades and the *E. coli* FdhF enzyme was among the first molybdoenzymes to be crystallized in 1997 [10], details of the reaction mechanism involving the first and second coordination sphere remained poorly understood. Furthermore, the catalytic mechanism of formate oxidation is still unclear [26]. As we have highlighted previously, the most promoted mechanisms proposed for FDH reflect a lack in clarity of the coordination environment and oxidation state of the bis-MGD cofactor. Overall, the formate oxidation mechanism is believed to be similar between Mo-containing and W-containing FDHs.

Early on in our own work, Tobias realized that metal-containing FDH enzymes and nitrate reductase active sites are surprisingly superimposable [27], with the latter harboring a Mo ion coordinated by the two characteristic MGD molecules, one terminal sulfido group and a cysteine sulfur atom [19]. In addition, the nitrate reductase active site comprises conserved threonine and methionine residues (arginine and histidine, in FDH, respectively). In the oxidized active sites of both enzymes, the Mo/W ion is hexacoordinated and a sulfur shift is needed to displace the selenocysteine or cysteine residue to create a vacant position for substrate binding (i.e., formate or nitrate) [28,29]. This is a mechanism that had been proposed by the Moura group [30].

Nitrate reductases catalyze the typical O atom transfer mechanism characteristic for most molybdoenzymes [31].

The similarities between FDH and nitrate reductases were highlighted by Tobias, who converted *R. capsulatus* FDH to an enzyme with nitrate reductase activity [27]. This was achieved by exchanging the histidine H387 for a methionine and the arginine R587 for a threonine, residues that are conserved in nitrate reductases. Further, an additional arginine was inserted into the active site of *R. capsulatus* FDH and this enzyme variant showed bis-MGD-dependent nitrate reductase activity. However, the involvement of the sulfido ligand of this enzyme variant in nitrate reductase activity was not clear in his work, since an enzyme form containing an oxo ligand instead of the sulfido ligand was also able to reduce nitrate [27]. Nevertheless, we had proposed a mechanism for nitrate reduction based on the results, stating that FDH can perform both oxygen atom transfer (for nitrate reduction) and C-H bond cleavage (for formate oxidation) [27]. This mechanism led to a lot of criticism in the field, since for formate oxidation, we were proposing the displacement of the cysteine residue by formate from the active site, based on our experimental data (iodoacetamide labelling and EXAFS studies). Furthermore, for nitrate reduction we were starting with the wrong oxidation state of the Mo ion (it was suggested that we talk to a chemist next time before proposing a mechanism). In our defense, I want to mention that these were only working mechanisms, which have usually the goal of being adapted when more conclusive data are available. Working mechanisms are supposed to encourage constructive discussions regarding experiments that can be carried out to prove or disprove any aspect of the mechanism. Unfortunately, no constructive criticism was received, only hints that we are wrong and the point of view of the others was correct. Admittedly, formate does not bind directly to the Mo, as described below, but likely interacts with a bound water instead.

Later, Hille and Niks proposed “the hydride-transfer mechanism” that, in their opinion, is based on experimental data [32,33]. This considers the binding of formate to the second coordination sphere without contacting the Mo ion directly [34]. In their proposal, the sulfido ligand abstracts a hydride ion from formate, resulting in a two-electron-reduced intermediate Mo^IV^− SH, and CO_2_ is released [35]. In this mechanism, however, the (seleno-) cysteine would not be involved and the role of the metal ion is only to provide a hydride acceptor by coordinating the sulfido ligand.

In the meantime, the Moura group was stepping back from the proposed sulfur-shift mechanism and was favoring the hydride transfer mechanism proposed by the Hille group [32]. However, the experimental data for this second coordination hydride transfer mechanism were only based on EPR studies, for one short-lived oxidation state in a coordination with six ligands at the Mo atom. There is no doubt that the sulfido ligand acts as hydride acceptor, which has been well-established for xanthine oxidoreductases [12]. Conclusively, to date, there has been no commonly accepted mechanism for metal-containing FDH enzymes. So far, the only evidence to speak against an oxygen atom transfer mechanism has been the report by Khangulov published in 1998 [36]. This showed that *E. coli* FdhF produces CO_2_ and not bicarbonate during formate oxidation using an experimental setup with ^18^O-labelled water and ^13^C-labelled formate, in which only ^13^C^16^O_2_ was observed as the initial product of the reaction. This experiment has not been questioned since and has led to the proposal of numerous mechanisms of how FDH catalyzes formate oxidation without considering any oxygen atom transfer (OAT) transitions [21,32].

## 7. The Mechanism of Formate Dehydrogenase: Classical Oxygen Atom Transfer or What Else?

In 2017, I started to reconsider the oxygen atom transfer mechanism and started to question the one experiment that had been performed to exclude the oxygen atom transfer mechanism. I was struck by the observation in several crystal structures that a water molecule had been identified next to the arginine in the second coordination sphere of the enzymes [15,37]. We had worked on variants of arginine and showed that this is the residue where azide, and likely also formate and CO_2_, are binding, since we obtained a competitive binding of azide, along with formate and CO_2_ binding at this site as substrates [22]. Luckily, I also received the support of a chemist, Carola Schulzke, who had the opinion that all molybdoenzymes have a common mechanism and that oxygen atom transfer is the most plausible reaction for formate oxidation involving the Mo atom. This view is consistent with very early studies on these enzymes. The class of Mo/W-containing enzymes were characterized initially in the 1980s by Holm and coworkers to catalyze classical oxygen atom transfer reactions [38], in which the oxygen atom from water is transferred to the substrate, or in the reverse reaction, from the substrate to produce water; these reactions are coupled to the reversible transfer of two electrons and two protons in the course of the transformation cycles [12]. The electrons are directly transferred to the Mo/W metal ion of the cofactor and the metals cycle between the Mo/W^IV^ and Mo/W^VI^ oxidation states, with Mo/W^V^ as a short-lived intermediate state. As has been noted above, FDH enzymes were, thus far, considered to be an exception in the group of Mo- and W-containing enzymes for not catalyzing an oxygen atom transfer reaction [27,32,35,36]. This exceptional behavior was mainly proposed based on the report by Khangulov in 1989 [36], using ^18^O-labelled water and ^13^C-labelled formate, in which no ^18^O was identified in the immediate product CO_2_. However, since the enzyme in the Khangulov experiment was inhibited by high amounts of azide (3 mM!) [36], which were used to slow down the reaction, we decided to reinvestigate the experiment in the absence of azide. In the meantime, a new postdoc had started in my lab, Hemant Kumar, who had experience with gas chromatography/mass spectrometry (GC–MS), the method used in the Khangulov experiment. We decided to repeat the experiment by using *R. capsulatus* FDH, an enzyme that we had characterized in detail previously and that, additionally, is more oxygen tolerant compared with the *E. coli* FdhF enzyme [7]. This enabled us to use low/no azide concentrations in the experiments. Instead, we performed the assay at 10 °C to slow down the secondary reaction of non-enzymatic CO_2_ hydration [39]. Our first results clearly showed that after a reaction time of 10 s with ^13^C-labeled formate, labelled ^13^C^18^O^16^O was detected, demonstrating that the oxygen of H_2_^18^O water is indeed inserted into the product, which is rather bicarbonate and not CO_2_. We showed that the enzyme-catalyzed reaction was much faster as compared to the secondary hydration of CO_2_ under our experimental conditions [40]. In contrast, in the azide-inhibited reaction, which we performed as a control, we obtained the same results as reported by Khangulov, showing that azide interferes with the reaction and with product formation. In the meantime, I started a collaboration with Carola Schulzke, whose group synthesized monothioformate and dithioformate, to analyze the reaction in more detail with a substrate the product of which is not so easily hydrated like CO_2_.

To further confirm the insertion of oxygen into H^13^COO^−^-labelled formate and to determine the first product formed during the reaction, we used NMR, in collaboration with Carola Schulzke’s group, as a second detection method to the GC–MS experiment, in which the bicarbonate was difficult to detect. For NMR using formate, the reaction was too fast, and the ^13^C-NMR measurements were relatively time intense, so we had to slow down the reaction with azide in this case. We performed the reaction at 5 °C to decrease the non-enzymatic hydration of CO_2_. The first product that was detected by NMR in substantial abundance was bicarbonate, the concentration of which already began to decrease before the formation of CO_2_ increased. The first data point, however, was drawn only after 25 min, which made it difficult to disentangle the still quite fast enzymatic reaction and the subsequent secondary reaction of the CO_2_/HCO_3_^−^ equilibrium at pH 9.0. Therefore, in order to obtain unquestionable data we used ^13^C-labelled thioformate in the experiment, in collaboration with Carola Schulzke. Thioformate has been shown to be a suitable substrate of *R. capsulatus* FDH in bi-substrate kinetic experiments performed in my lab. When ^13^C-labelled thioformate was used in the NMR experiment with low azide concentrations, thiocarbonate was clearly detected as the first intermediate before COS abundance began to increase steadily. Here, thiocarbonate is detected instead of thiobicarbonate, based on the fact that thiobicarbonate is more acidic than bicarbonate, less stable at this pH and is, in fact, easily deprotonated [40]. This clearly confirmed the oxygen atom transfer with bicarbonate/thiocarbonate as first reaction intermediate of the reaction, which are released from the enzyme before CO_2_ or COS are formed in the slower non-enzymatic secondary reaction outside the enzyme. Surprisingly, in a publication that was published before our work, the Hille and Raman groups also showed the formation of bicarbonate before CO_2_ production by NMR; however, they did not add any mechanistic comments or considerations to this result in their publication [41].

**Figure 2:**
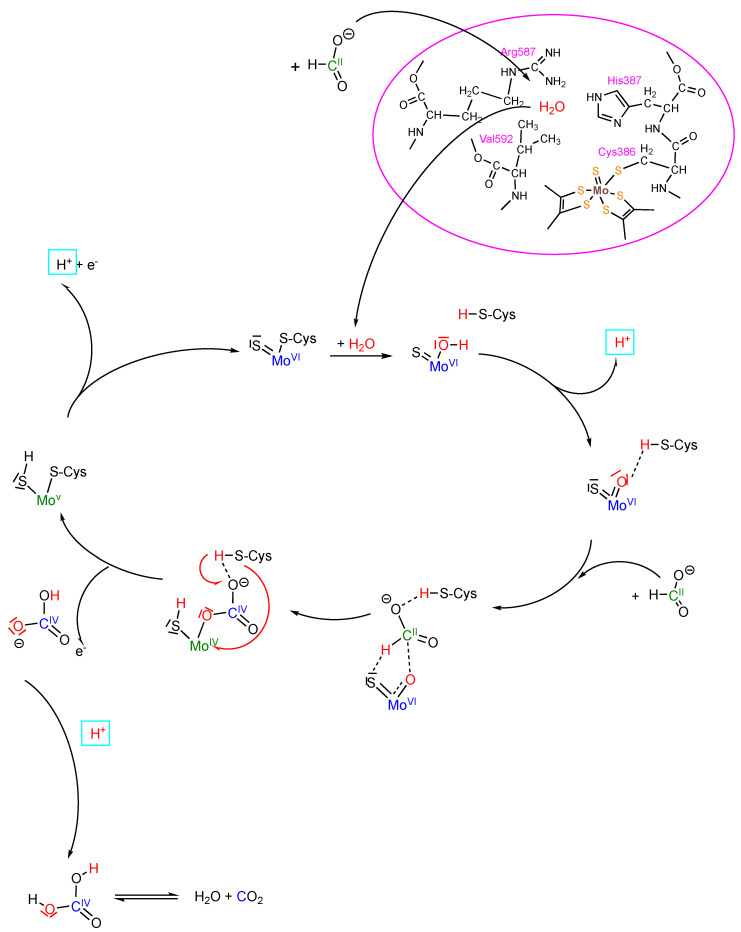
Proposed oxygen atom transfer mechanism of formate oxidation by a Mo-Cys-containing formate dehydrogenase. Details of the mechanism are given in the text.

Additionally, we also investigated the back reaction of CO_2_ reduction to clarify whether bicarbonate enters the enzyme or whether CO_2_ does instead. This was followed by bicarbonate formation in the enzyme environment at the active site to enable the oxygen atom transfer mechanism. Using ^12^CO_2_-saturated buffer in the presence of H_2_^18^O-saturated enzyme, we indeed obtained H^12^C^18^O^16^O^−^-labelled formate in the first 10 s of the reaction; thus, the results showed that CO_2_ is the primary substrate that enters the enzyme, which is then likely converted to H^12^C^18^O^16^O_2_ bicarbonate at the active site (possibly in a carbonic-anhydrase-like hydration reaction) that is then used as a direct substrate for the reduction to formate and water. When H^13^CO_3_^−^ was used instead in the reaction, no ^18^O-labelled formate was obtained. It is likely that bicarbonate is not used as a direct substrate in the back reaction, since it cannot enter the enzyme through the hydrophobic CO_2_ channel. In the NMR experiment using ^13^C-labelled formate, bicarbonate was detected as the first intermediate product. It was detected in an abundance and was much higher than the enzyme concentration, suggesting that bicarbonate is released from the enzyme and not CO_2_. Could this suggest that bicarbonate can be released from the enzyme but not enter it? We proposed that after reduction of the enzyme with formate, conformational changes in the second coordination sphere occur. Indeed, in the crystal structures of the *D. vulgaris* FDH enzyme [37] and the reinterpreted structure of the *E. coli* FdhF enzyme [42], and the structural rearrangements of the formate-reduced enzyme (in particular by movement of the histidine residue), the formate tunnel becomes accessible for the release of bicarbonate. This is an excess site that is blocked in the oxidized enzyme or azide inhibited enzymes. Indeed, bicarbonate cannot enter the active site through the formate channel. We assume that the binding site for CO_2_ hydration and bicarbonate dehydration is at the conserved arginine residue in the second coordination sphere, since in the crystal structures of the *D. vulgaris* [37] and formylmethanofuran dehydrogenase from *Methanothermobacter wolfeii* [15], a water molecule was identified to be bound in vicinity to this residue. In previous studies, it has also been shown that azide is bound to this residue in the active site fitting and our hypothesis stated that this is the competitive binding site for azide and CO_2_/bicarbonate [22,43]. Overall, our results led to the proposal by Carola Schulzke and myself of an oxygen atom transfer mechanism for the reversible oxidation of formate by metal-containing FDH enzymes, as shown in Figure 2 [40].

The catalytic cycle starts with the oxidized active site Mo^VI^ coordinated by one sulfido and one cysteinate ligand. Water is bound in the second coordination sphere at the arginine in the active site, and upon the entrance of formate, the water is displaced from this site accompanied by some slight structural changes that result in displacement of the cysteinate and coordination of water to the Mo. This water is instantly deprotonated due to the increased acidity induced by the highly oxidized Mo and one proton is transferred to the cysteine sulfur. Essentially this is an insertion of -O–H into the Mo-S(Cys) bond. The second water proton is also detached due to the high acidity and becomes part of the proton pool. The resultant active site is a Mo^VI^OS core that is stabilized by hydrogen bonding to cysteine. Formate is inserted into the hydrogen bonding between the active site complex and cysteine, with the formate hydrogen interacting with the sulfido and oxido ligands and formate oxygen interacting with the cysteine proton, again resulting in a hydrogen bonding stabilized species. The concomitant sulfido/oxo coordination has yet to be identified, but can be expected to be short-lived. Through a short-lived transition state in which the formed C-H and the Mo bonds are weakened and the S-H and C-O bonds have already induced electrons or electron pairs, this begins to turn in a circular motion. The formate hydrogen is transferred to the sulfido ligand; whether this is more reminiscent of a hydride transfer to sulfur or of a proton transfer to sulfur remains open in the proposed mechanism. This results in a species with a reduced Mo^IV^ center and an oxidized carbon (IV) product. The cysteinate-bound proton is then transferred to carbonate forming bicarbonate, and the cysteine thiolate in turn binds to the Mo, thereby displacing the product. Upon product release as bicarbonate and an electron transfer to the first FeS cluster, the Mo^V^ as an intermediate is formed in a six-coordinated state. After proton and electron release, the initial oxidized Mo^VI^ state is formed again, ready for a new turnover. The bicarbonate is released from the enzyme and its subsequent dehydration to CO_2_ likely occurs outside of the enzyme environment. The key step of this proposed mechanism constitutes a common oxygen atom transfer mechanism. This mechanism is in accordance with all undisputed experimental data available, and is stoichiometrically balanced and hence coherent.

In our mechanism, we propose we that after formate binds the amino acid ligand at the Mo atom, it is displaced by water. The displacement of the cysteine ligand in *R. capsulatus* FDH has been demonstrated by us in previous studies via iodoacetamide labelling of the nitrate-inhibited and formate-reduced enzyme, and in addition, by EXAFS studies [19,27]. The iodoacetamide labelling of the selenocysteine ligand has also been shown for the *E. coli* enzyme [27]. EXAFS studies of the *R. capsulatus* enzyme further proved the displacement of the cysteine residue in the formate-reduced enzyme by an oxygen atom (which could be the one from water, as shown in this study). In the EXAFS data of the azide or cyanate-inhibited enzyme, instead of cysteine sulfur, a light atom was observed as being bound to the Mo center [13]. We assigned this as an oxygen atom from water instead of a C or N atom from the inhibitors, which have been proposed as not directly binding to the Mo. The binding of water observed in the EXAFS studies would support our oxygen atom transfer mechanism [13]. Often, results from EPR studies are considered as an argument for the oxidation-state-dependent active site structure of the enzyme. However, the Mo^V^ active site constitutes the intermediate state after product release and one electron oxidation, in which the amino acid ligand is quite likely rebounded again to the Mo atom which would otherwise be coordinatively unsaturated [35]. Therefore, the EPR data were in accordance with our mechanism. In previous studies, several groups proposed a hydride transfer mechanism with the formate being bound within the second coordination sphere of the active site metal [44]. One of the arguments used by the authors to support their mechanism was that the second p*K*_a_ value of formic acid (i.e., the one for C-H dissociation) disfavors a proton abstraction, and the resulting carbanion that is formed would be unstable. In our mechanism, the formate is directly bound to the Mo atom through an oxido function derived from a water molecule, so that a carbanion would not be formed after proton abstraction [32]. Nevertheless, in our mechanism we have left it open as to whether the hydrogen atom of formate is transferred as a hydride or in a proton-coupled electron transfer reaction to the sulfido ligand, forming the SH group and the reduced Mo^IV^ (i.e., we do not propose the direction in which the electrons or electron pairs move when entering the transition state).

Some other groups, like Meneghello et al. [45], chose an electrochemical approach with the W-containing FDH from *Desulfovibrio vulgaris* Hildenborough for confirming the substrate of FDHs for the back reaction of CO_2_ reduction as being CO_2_ rather than HCO_3_^−^, in their opinion. As has already been emphasized by Cooper et al. [39] and confirmed by our investigation, these studies do not necessarily reflect what is happening directly at the active site. We therefore propose that for the reduction of CO_2_, the intermediate ionic species HCO_3_^−^ is hindered from entering the active site via the hydrophobic CO_2_ channel that has been identified in crystal and cryogenic EM structures, while CO_2_ can easily enter. This is still consistent with the report by Meneghello et al. [45]. At the active site, CO_2_ then reacts with H_2_O to form HCO_3_^−^ (in a carbonic-anhydrase-like reaction) which subsequently provides the direct substrate for the back reaction resulting in formate production. Therefore, by electrochemical studies, one cannot measure what is directly happening at the active site of the enzyme. In conclusion, to confirm the oxygen atom transfer mechanism and the carbonic anhydrase activity at the active site, more investigations are required in the future to provide a conclusive mechanism that is accepted in the field. My definitive suggestion to people working on mechanistic studies of metal-containing FDH enzymes is to work in the absence of an inhibitor and to work under strictly anaerobic conditions.

## 8. Formate-Dehydrogenase-Catalyzed CO_2_ Reduction

Undoubtedly, the fixation and utilization of carbon dioxide by living organism is a difficult task. This problem is also made obvious by the reaction of the D-ribulose-1,5-bisphosphate carboxylase/oxygenase (RuBisCO), with its promiscuous (CO_2_ versus O_2_, energy wasting) and slow (<10 s^−1^) catalytic performance [46]. CO_2_ reduction using FDH enzymes naturally occurs by acetogens, which grow by converting CO_2_ to acetate by syntrophs, which produce formate and H_2_ as vehicles for interspecies electron transfer, and finally, by the closely related sulfate-reducing bacteria, which in the absence of sulfate, also turn to a syntrophic lifestyle [7,32,35,47]. In fact, the enzymes with the highest CO_2_-reducing activities are all derived from these organisms, and include the hydrogen-dependent CO_2_ reductases (HDCR) from the acetogens *Acetobacterium woodii* and *Thermoanaerobacter kivui* [48,49,50], the W-FDH from the syntroph *Syntrophobacter fumaroxidans* [51,52], and the Mo-FDH from the sulfate reducer *Desulfovibrio desulfuricans* [47,49]. Further, the enzymes from *R. capsulatus*, *C. necator*, *E. coli*, *Methylobacterium extorquens*, *Rhodobacter aestuarii* and *Methanococcus maripaludis* have been described as catalyzing both the reaction of formate oxidation and CO_2_ reduction in solution assays using purified enzymes [7,21,25,53,54,55,56]. Mostly, a high concentration of an electron donor had to be used to achieve the unfavored back reaction of CO_2_ reduction, in concentrations that would usually not be present in the cell.

The two W-FDHs of the anaerobic syntrophic *S. fumaroxidans* [52] were shown to interconvert formate and carbon dioxide. FDH1 was demonstrated to be an efficient carbon dioxide reductase, with a higher rate for carbon dioxide reduction than for formate oxidation; In addition, the acetogenic *Morella thermoacetica* was shown to have CO_2_ reductase activity, but, in these cases, was dependent on NAD(P)H [57]. Notably, the reduction of CO_2_ with NAD(P)H (either by W-FDH or Mo-FDH) is thermodynamically quite unfavorable, with reduction potentials of −0.43 and −0.32 V, respectively. This thermodynamic constraint highlights the key role played by these enzymes in overcoming the reaction energy barrier, allowing those organisms to effectively reduce CO_2_ to formate. Other acetogens, such as *A. woodii*, developed a specific and remarkable hydrogen-dependent carbon dioxide reductase complex that couples the carbon dioxide reduction directly with the dihydrogen oxidation. This notable reductase complex allows CO_2_ reduction by dihydrogen with a *k*_cat_ of 28 s^−1^, even though the reverse reaction (formate oxidation and dihydrogen formation) is 1.4 times faster. Although the W-FDHs are suggested to be more efficient at reducing carbon dioxide than the Mo-FDH counterparts, because of the lower reduction potential of W^IV^ compared to Mo^IV^, different Mo-dependent CO_2_ reductases have been described. However, these do not act as such enzymes under cellular conditions [35]. For example, the Mo/NAD^+^-FDH of *Cupriavidus oxalaticus* has long been known to be able to reduce carbon dioxide, although in a reaction that is ≈30 times slower than the formate oxidation [58]. The *R. capsulatus* Mo-FDH is also able to catalyze both formate oxidation and CO_2_ reduction in solution, but the CO_2_ reduction reaction is ≈20 times slower than formate oxidation [7].

**Figure 3:**
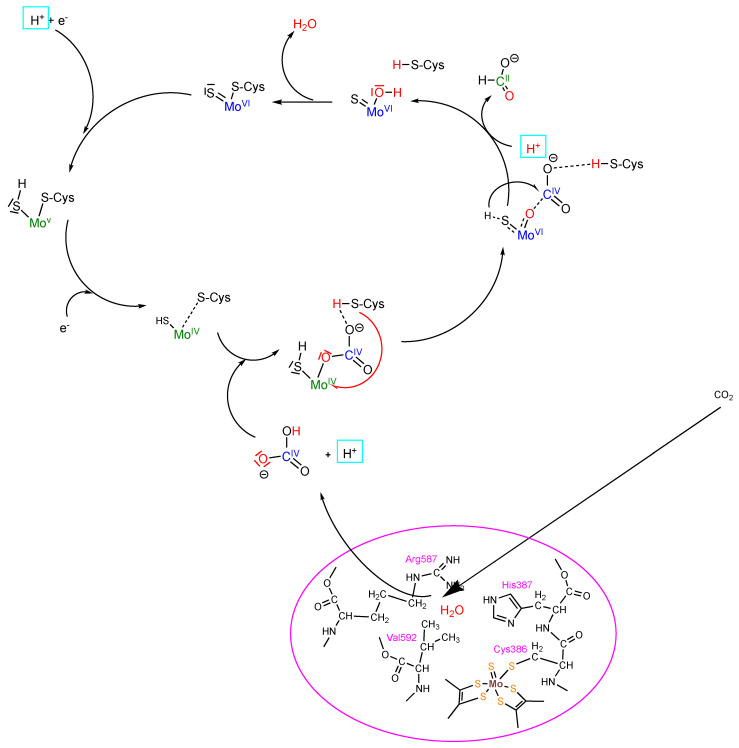
Proposed mechanism for CO_2_ reduction by Mo-Cys-containing formate dehydrogenase. Details of the mechanism are given in the text.

The reaction of CO_2_ reduction is the reverse of formate oxidation, as shown in Figure 2. As has been explained above, CO_2_ enters the enzyme by the hydrophobic CO_2_ channel (as revealed in the cryo-EM structure [8]) and binds at arginine 587 (numbering of *R. caps* FDH), where a water is also bound (as shown in the crystal structures of [15,37]) (Figure 3). Via carbonic-anhydrase-like activity, bicarbonate is formed, which then can be bound to the reduced Mo^IV^ ion at the active site by displacing the amino acid ligand. After a further proton transfer, formate is released, and water is formed and released in the next step, yielding the oxidized Mo^VI^ state (Figure 3). This mechanism was revealed by the addition of H_2_^18^O and ^12^CO_2_ (in the presence of ^13^C-labeled bicarbonate), which gave rise to only ^18^O^12^C-labeled formate.

## 9. Final Conclusions and Future Perspectives

In my opinion, I have presented conclusive data for the new oxygen atom transfer mechanism for metal-containing FDH enzymes, proposed by Carola Schulzke and myself. Since we are revising an established dogma in the field, we were expecting it to be difficult to convince other researchers to reconsider their favorite mechanism that they have been working on for many years. Of course, data should speak for themselves and in the event that other people have convincing data to show that we are wrong, we will accept this. As Einstein can be cited as saying: “No amount of experimentation can ever prove me right; a single experiment can prove me wrong.” I will end here and hope for a bright future with new researchers entering the field to bring in new ideas and perspectives.

## Data Availability

No new data were generated for this review.
